# Effective coverage of child immunisation service in Ethiopia

**DOI:** 10.1038/s41598-025-02885-0

**Published:** 2025-05-29

**Authors:** Samrawit Birhanu Alemu, Aynalem Belay, Redait Awoke Assefa, Melaku Birhanu Alemu

**Affiliations:** 1 Department of Public Health, Debre Markos University, Debre Markos, Ethiopia; 2 Department of Midwifery, Wolkite University, Wolkite, Ethiopia; 3 School of Medicine, College of Medicine and Health Science, University of Gondar, Gondar, Ethiopia; 4 Department of Health Systems and Policy, Institute of Public Health, University of Gondar, Gondar, Ethiopia; 5 Curtin School of Population Health, Curtin University, Perth, WA Australia

**Keywords:** Health policy, Health services

## Abstract

Vaccines are one of the safest and most cost-effective interventions to prevent disease and disabilities worldwide. Yet, nearly 19 million children do not receive basic vaccines and most of them are from low and middle-income countries. The Sustainable Development Goal 3 aims at eliminating preventable deaths of newborns and under-five children by 2030. However, Ethiopia has a high child mortality rate, and most of them are from preventable causes. Quality provision of immunisation could reduce vaccine-preventable morbidity and mortality. Despite this, the quality-adjusted coverage of immunisation services in Ethiopia has not been well-documented. We used the Kids’ record from the 2019 Ethiopian Demographic and Survey (EDHS) and the health facility recode from the 2021–2022 Ethiopian Service Provision Assessment (ESPA). The EDHS and ESPA data were linked using administrative boundary linkage using administrative regions and city administrations. The crude coverage was weighted before linking with the ESPA dataset. Health facilities which provide child immunisation services were included. The quality of immunisation service provision was constructed from 14 indicators. The quality of immunisation was calculated as the sum of the availability of items in each domain, divided by the total number of items in the domain, and finally multiplied by 100. The effective coverage was measured as the product of contact coverage and quality of immunisation service provision. We use STATA 17 for analysis. A total of 951 respondents who have a live children aged 12–23 month were included. The national effective coverage of immunisation was 34.3% (95% Confidence interval (CI): 33.0, 35.5) with a large variation in different regions, ranged from 14.8% in Afar to 83.5% in Addis Ababa. The crude coverage of immunisation services was 39.6% (95% CI: 36.5, 42.7), ranged from 19.2% in Afar to 87.39% in Addis Ababa. The overall score for quality of immunisation service was 82% (95% CI: 80.44, 83.51). The quality ranged from 74.6% in SNNPR to 95.5% in Addis Ababa. The quality of immunisation varies with the managing authority of health facilities, private facilities had the highest quality (90% (95% CI: 85.35, 95.36)) and government facilities scored the lowest quality (82% (95% CI: 80.06, 83.22)). There was low effective coverage of immunisation services provision in Ethiopia. The effective coverage was lower in the pastoral regions (Afar and Somali). Government facilities and facilities in rural areas had lower-quality immunisation services. Facilities in SNNPR and Afar region had low-quality of immunisation service. Policymakers should focus on improving the quality of government facilities and the facilities in rural areas and pastoral regions to improve immunisation services.

**Supplementary Information:**

The online version contains supplementary material available at 10.1038/s41598-025-02885-0.

## Introduction

Expanded Program on Immunisation (EPI) is one of the most important public health interventions used to reduce child morbidity and mortality associated with infectious diseases^1^. It forms a key building block for the healthcare delivery systems by which globally, an estimated 3 million deaths are prevented by vaccination each year through its successful implementation^1,2^. Vaccines are one of the safest and most cost-effective interventions and vaccination coverage is mentioned as part of Sustainable Development Goal 3 which aims at eliminating preventable deaths of newborns and under five children by 2030^3^. Despite its importance, currently, 19 million children do not receive basic vaccines and of this, 60% are from low and middle-income countries^5,6^.

In Ethiopia, two out of ten (19%) children aged 12–23 months had not received any vaccines^7^. Ethiopia is among the countries with the highest rates of vaccine-preventable diseases (VPDs), contributing significantly to child mortality rates and 16% of under-five mortality in Ethiopia is attributed to vaccine-preventable diseases, such as measles, pneumonia, and diarrheal diseases, which could be prevented with timely vaccinations^8–10^. According to the 2016 Ethiopian Demographic Health Survey, 39% of children aged 12–23 months were vaccinated, with rates varying greatly across geographical regions and only 22% were vaccinated at the appropriate age^10–12^. Immunisation coverage in Ethiopia was 44% in 2019, referring to children who received the full schedule of basic vaccines (BCG, DPT, polio, and measles), ranging from the highest in Addis Ababa (83%) and Afar (20%).

To improve immunisation coverage in Ethiopia, interventions such as implementing an integrated health extension package, training health extension workers, and expanding primary healthcare services were carried out^13^. Access to quality healthcare includes different factors, such as availability, acceptability and affordability^14,15^. Numerous studies had revealed that low immunisation coverage can be attributed to a variety of factors, including family socioeconomic and demographic characteristics, access to maternal health services, knowledge of immunisation, and maternal satisfaction. Each of these factors plays a crucial role in enhancing the quality of immunisation^16–19^.

The quality of immunisation service depends on the service accessibility, waiting time and, availability of vaccines^20^. In Ethiopia, one of the EPI strategy objectives is to improve the quality of child immunisation^21^. Accordingly, knowledge of the benefits of child vaccination, and the health facilities’ readiness to provide the vaccination service is important to improve the quality of immunisation service^22^. To ensure the quality of immunisation of vaccine safety, which is proper handling and storage, safe vaccine administration (trained staff) and safe waste removal are required^23^.

Effective coverage is one of the approaches which can be used to monitor health system performance by including the use, needs and quality of healthcare services^24^. Although there are ways to measure effective coverage for health care services, quality-adjusted coverage is preferable to measure maternal and child health services as it has the potential to provide an overall estimate of effective coverage in global, national, and local settings^25^.

This study was conducted to assess the effective coverage of child immunisation in Ethiopia. To our knowledge, it is the first study on the effective coverage of immunisation in Ethiopia. The findings will help track the quality of immunisation and the performance of the health system, contributing to improvements in the overall effectiveness of healthcare delivery.

## Methods

### Study area

Ethiopia is a federal state located in the horn of Africa which is divided into 9 sub-regions and two city administrations. Ethiopia has three tire health systems (primary, secondary and tertiary). The primary level of care includes primary hospitals which provide emergency surgical services, including caesarean sections, and blood transfusion services, health centers which serve an average population of 100,000 people and five health posts in each health center catchment area. The secondary level contains the general hospital which provides inpatient and ambulatory services and patients referred from primary hospitals. The last tier system is the tertiary level which encompass the specialized hospitals that serves an average of 5 million people including referral from general hospitals^26^.

### Data source

In this study, we have linked two nationally representative community based and facility-based datasets. The Ethiopian Demographic and Health Survey (EDHS) 2019 provided individual-level data on immunisation coverage across the population, offering insights into vaccination rates and coverage patterns at the individual level, allowing for a detailed analysis of coverage rates at both regional and national levels. The other dataset used was SPA (Service Provision Assessment) which is part of the DHS program and SPA 2020–2021 is a facility-based survey which collects data on quality of care and service availability. This dataset includes records from health facilities, detailing factors such as the availability of vaccines, quality of healthcare services, and adherence to clinical guidelines. By examining the health facility records from ESPA, we were able to assess the quality of health services provided, which allowed us to calculate and interpret key indicators of healthcare quality. By combining individual immunisation data from EDHS 2019 with facility-based quality metrics from ESPA 2021–2022, we created a comprehensive assessment of both coverage and quality of immunisation services in Ethiopia. The EDHS holds data that is publicly available via DHS at https://dhsprogram.com/.

### Measure of variable

#### Coverage variable

Child immunisation was recorded based on the information from their immunization card and the mother’s report. A child was considered fully immunized if either the card was available or the mother provided confirmation^27^. Full child immunisation was defined as receiving all essential vaccines with in the age group of 12–23 months. A child is considered fully immunised if they received one dose of BCG vaccine for tuberculosis, three doses of the pentavalent vaccine (DPT-HepB-Hib), three dose vaccines for polio and one dose of measles^7^.

#### Quality of care variables

There are three dimensions used to measure the quality of service, these are structure, process, and outcome^28^. We use the structural component of child immunisation service to assess the quality which, refers to the facility’s readiness to give immunisation services including the availability of trained personnel, equipment, medical supplies and commodities^29^. We used 14 indicators to generate the quality of health facilities (Supplementary Table 1).

Child immunisation service readiness scores were calculated using averages based on the WHO guideline^30^. The readiness of immunisation had three domain (staff and guideline, equipment and, medicines and commodities), Staff and guidelines variable staff were recoded as “yes” if the staff were trained and “no” if they did not receive any training. Guidelines were recorded as “yes” if observed and “no” if none were available or reported but not observed. Equipment variables were coded as “yes” if they were observed and valid and else recoded as “no”. Medicines and commodities variables were recoded as “yes” if they were observed and at least one valid and else recoded as “no”^25^. The inactivated polio vaccine was excluded from the study due to the absence of records in the ESPA dataset.

The readiness score was calculated as a sum of the available items of each domain, divided by the total number of items in the domain, finally multiplied by 100^30^.

$$\:Quality\:\left(readiness\right)=\frac{sum\:of\:avilable\:items}{total\:number\:of\:items}\:*100\:$$

#### The sociodemographic variables

We have used sociodemographic variables to describe the participants which were extracted from the kids’ record (KR) of the EDHS 2019 dataset. The residence was classified as urban and rural. Women’s education was classified as no education, primary, secondary and higher. Wealth index was categorized into five levels (poorest, poor, middle, rich, and richest). These scores were assigned based on the quantity and types of consumer goods a household possesses, such as a television, bicycle, or car, along with housing features like the source of drinking water, toilet facilities, and flooring materials^29^. We have recoded age, marital status and religion as literatures. Mother’s age were classified as “15–24”, “25–35” and “36–49”^33^. Marital status was recorded as currently married and unmarried (single /divorced /separated/widowed)^34^. Religion was recoded as Orthodox, Muslim, Protestant and Other (Catholic, traditional and other)^33^.

#### Data management and analysis

The data was cleaned, and missing values were managed using a guide to DHS statistics. The data were weighted using sampling weight to restore the representativeness of the survey.

To determine immunisation coverage, we utilized the Kids Record (KR) file from the Ethiopia Demographic and Health Survey (EDHS), focusing on a sample of 1,028 children aged 12–23 months who were a live at the time of the survey. However, to maintain data consistency, we excluded the Tigray region from our analysis due to the absence of corresponding records in the Ethiopia Service Provision Assessment (ESPA) dataset. Accordingly, by removing 93 observations from Tigray, the final unweighted sample size was 915. For the quality measurement, we used the health facility records from the Ethiopia Service Provision Assessment (ESPA) dataset, which includes data from 1,158 health facilities across the country. For our analysis, we selected 774 facilities that specifically provide immunization services to ensure our focus aligned with relevant healthcare delivery points.

We have used administrative boundary linkage method to link the two datasets^31,32^. First, we calculated the proportion of immunisation coverage for each region, enabling us to match the regional average coverage with facilities located within the same area. This matching process facilitated a region-specific analysis of immunisation coverage across healthcare facilities, thereby enhancing the accuracy of the coverage estimates. This approach provided a clearer overview of quality-adjusted immunisation coverage across available regions in Ethiopia, aligning with the data completeness of the ESPA dataset.

The effective coverage of immunisation was calculated by multiplying the quality of the health facility (readiness) with the immunisation coverage. The formula was adopted from the WHO effective coverage framework^25,33^.

Effective coverage = Quality of the health service * crude coverage.

We use Stata 17 for analysis. Descriptive statistics was done to describe the socioeconomic and demographic characteristics of respondents and the facility characteristics. Additionally, sensitivity analyses were conducted to evaluate the impact of heavily skewed regions on the effective coverage of child immunisation. Finally, the sociodemographic characteristics of respondents and the characteristics of the facilities were presented using tables and graphs.

## Results

The study included 951 women with live children aged 12–23 months. Among them, 30.4% were aged 15–24, 56.8% were 25–35, and 12.7% were 36–49 years old. Additionally, the majority of participants (69.5%) resided in rural areas. (Table 1).

**Table 1:** Socioeconomic and demographic characteristics of participants EDHS 2019 (*n* = 951).

Variables		Frequency	Percentage
Mother’s age	15–24	290	30.47
	25–35	540	56.81
	36–49	121	12.72
Marital status	Unmarried	37	3.94
	Married	913	96.06
Residence	Urban	290	30.48
	Rural	661	69.52
Religion	Orthodox	309	32.46
	Muslim	348	36.57
	Protestant	265	27.9
	Others	29	3.07
Region	Afar	15	1.58
	Amhara	218	22.91
	Oromia	405	42.62
	Somali	56	5.88
	Benshangule	11	1.12
	SNNPR	199	20.96
	Gambela	4	0.44
	Harari	3	0.27
	Addis Abeba	34	3.60
	Dire Dawa	6	0.63
Mother’s education	No education	433	45.51
	Primary education	395	41.59
	Secondary education	73	7.65
	Higher	50	5.25
Wealth status	Poorest	214	22.5
	Poorer	169	17.74
	Middle	221	23.23
	Richer	168	17.7
	Richest	179	18.84

SNNPR: Southern Nations, Nationalities and Peoples.

### Health facility characteristics

A total of 774 health facilities that provide immunisation services were included. A large proportion of the health facilities were from rural areas (58%), and most of health facilities were hospitals (35%) (Table 2).

**Table 2:** Health facilities characteristics across regions in Ethiopian SPA 2021–2022 (*n* = 774).

Region	Facility	Facility type (*N* (%))				Area (*N* (%))		Managing authority (*N* (%))		
		H	HC	HP	C	Urban	Rural	Gov	Priv	NGO
Afar	34	4 (11.76)	15 (44.12)	15 (44.12)	N/A	10 (29.41 )	24(70.59)	34 (100)	N/A	N/A
Amhara	122	59 (48.36)	33(27.05)	30 (24.59)	N/A	61 (50.00)	61 (50.00)	121 (99.18)	1 (0.82)	N/A
Oromia	151	78 (51.66)	41(27.15)	32 (21.19 )	N/A	69 (45.70)	82 (54.30)	148 (98.01)	N/A	3 (1.99)
Somali	65	12 (18.46 )	26 (40.00 )	26 (40.00 )	1(1.54)	21 (32.31)	44 (6.69)	64 (98.46)	N/A	1 (1.54)
Benshangule	36	5 (13.89)	15 (41.67)	15 (41.67)	1(2.78)	8 (22.22)	28 (77.78)	35 (97.22)	N/A	1 (2.78)
SNNPR	211	80 (37.91)	60 (28.44)	69 (32.70 )	(0.95)	68 (32.23)	143(67.77 )	205 (97.16)	2 (0.95)	4(1.90)
Gambela	38	5 (13.16)	19 (50.00)	14 (36.84)	N/A	12 (31.58)	26 (68.42)	37 (97.37)	N/A	1 (2.63)
Harari	32	4 (12.50)	8(25.00)	20 (62.50)	N/A	14(43.75)	18(56.25)	31 (96.88)	1 (3.13)	N/A
Addis Abeba	43	22 (51.16)	21 (48.84)	N/A	N/A	43(100)	N/A	30 (69.77)	13(30.23)	N/A
Dire Dawa	42	5 (11.90)	15 (35.71)	21 (50.00)	1(2.38)	16(38.10)	26 (61.90)	38(90.48)	3 (7.14)	1 (2.38)
National level	774	274 (35.40)	253 (32.69)	242 (31.27)	5 (0.65)	322 (41.60)	452 (58.40)	743 (95.99)	20 (2.58 )	11 (1.42)

SNNPR: Southern Nations, Nationalities and Peoples; N: Number of health facility; H: Hospitals; HC: Health center; HP: Health post; C: Clinic GOV: Government; Priv: Private; NGO: Non Government Organisation; N/A: None Available.

### Crude coverage of immunisation

**Fig. 1:**
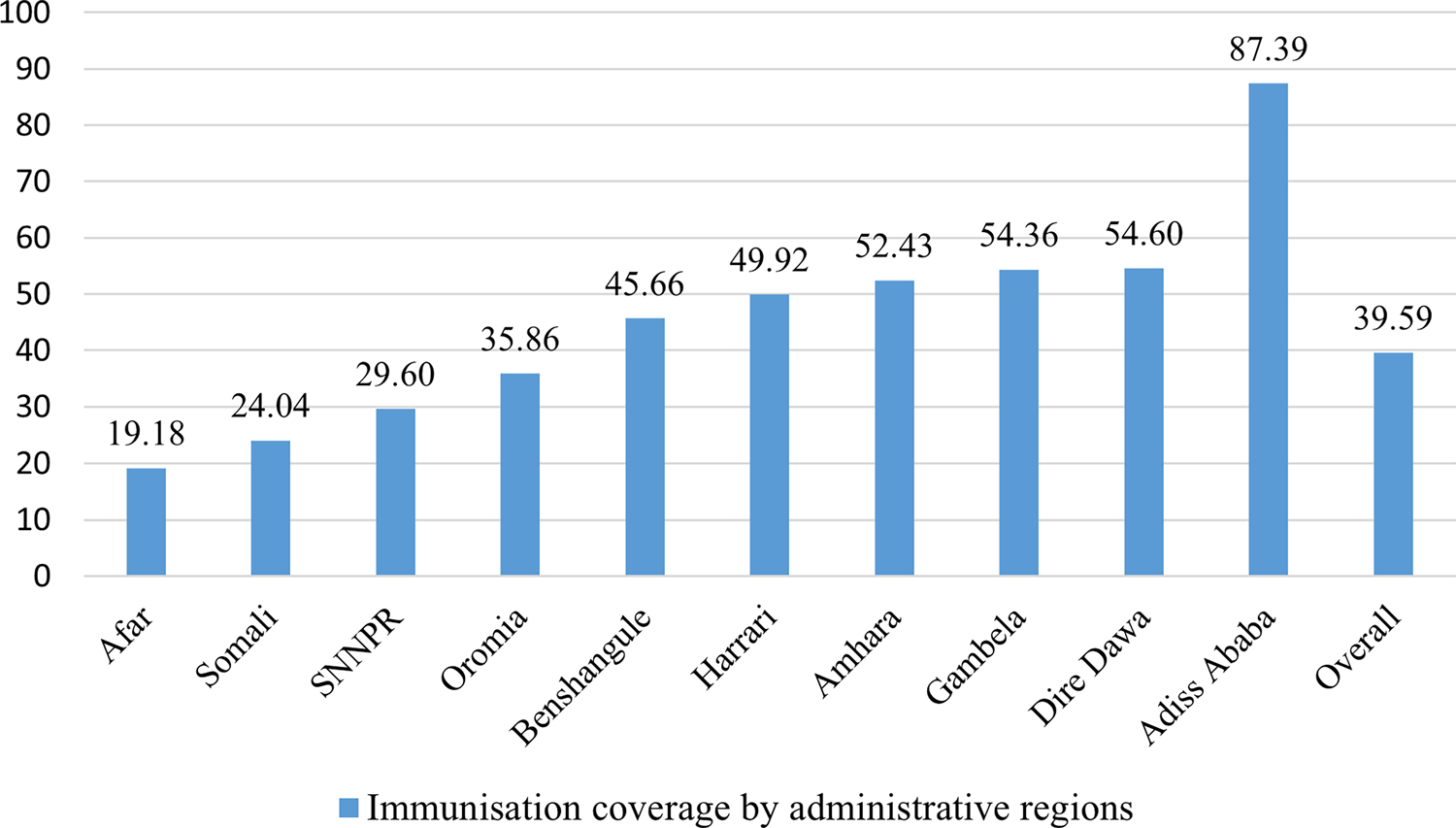
Crude coverage of immunisation across administrative regions.

The crude coverage of immunisation services in Ethiopia was 39.59% (95% CI: 36.47, 42.70), ranges from 19.18% (95% CI: 0.00, 41.74) in Afar to 87.39% (95% CI: 75.67, 99.12) in Addis Ababa (Fig. 1).

### Quality

A total of 43.38% (95%CI: 41.60, 45.16) of health facilities, with a regional variation from 36.37% (95%CI: 32.66, 40.09) in the Southern Nations, Nationalities, and Peoples’ Region (SNNPR) to 61.63% (95%CI: 56.49, 66.76) in Addis Ababa, were equipped with adequate staff and guidelines. Additionally, 79.15% (95%CI: 76.46, 81.84) of health facilities had sufficient medicines and commodities, with the lowest availability in Afar at 67.65% (95%CI: 51.90, 83.39) and the highest in Addis Ababa at 92.56% (95%CI: 86.11, 99.00). Furthermore, 87.71% (95%CI: 86.58, 88.83) of facilities are equipped with necessary medical equipment, ranging from 81.72% (95%CI: 79.48, 83.96) to 97.67% (95%CI: 95.77, 99.57) in Addis Ababa (Table 3).

**Table 3:** Quality of health facilities in each domain by regional Ethiopian SPA 2020–2021.

Region	Domain 1: Staff and guideline (95%CI)	Domain 2: medicine and commodities (95%CI)	Domain 3: equipment (95%CI)	Quality (95%CI)
Afar	43.38 (35.55, 51.22)	67.65 (51.90, 83.39)	84.87 (77.94,91.81)	77.31 (67.94,86.69)
Amhara	41.60 (37.42, 45.78)	83.11 (76.73, 89.50)	91.80 (89.39, 94.22)	85.42 (81.93,88.91)
Oromia	42.22 (38.09, 46.35)	80.53 (74.45, 86.61)	87.04 (84.62, 89.46)	81.79 (78.37, 85.21)
Somali	44.23 (38.36, 50.10)	80.31 (71.18, 89.43)	88.13 (83.51, 92.75)	82.53 (76.97, 88.08)
Benshangule	52.78 (46.45, 59.10)	88.89 (79.96, 97.81)	94.05 (89.23, 98.86)	91.27 (85.82, 96.72)
SNNPR	36.37 (32.66, 40.09)	71.66 (65.83, 77.48)	81.72 (79.48, 83.96)	74.61 (71.42, 77.81)
Gambela	49.34 (41.35, 57.33)	83.68 (72.57, 94.80)	87.22 (81.57, 92.87)	84.4 (77.82, 90.97)
Harari	56.25 (48.32, 64.18)	88.13 (78.81, 97.44)	94.64 (91.24, 98.04)	90.63 (86.17, 95.08)
Addis Abeba	61.63 (56.49, 66.76)	92.56 (86.11, 99.00)	97.67 (95.77, 99.57)	95.51 (92.52, 98.51)
Dire Dawa	44.64 (37.86, 51.42)	74.76 (61.41, 88.11)	89.46 (85.28, 93.63)	81.97 (75.30, 88.65)
National level	43.38 (41.60, 45.16)	79.15 (76.46, 81.84)	87.71 (86.58, 88.83)	81.98 (80.44 83.51 )

SNNPR: Southern Nations, Nationalities and Peoples.

The overall score for quality of immunisation service is 82% (95% CI: 80.44,83.51). The quality ranges from 74.6% (95% CI: 71.42,77.81) in SNNPE to 95.5% (95% CI: 92.52,98.51) in Addis Ababa. The quality of immunisation also varies with the type of health facilities, clinics had the highest quality (91.4% (95% CI: 79.86,100)) while health posts had lower quality 64.1%(95% CI: 60.78,67.50) (Table 4).

**Table 4:** Quality of health facilities by facility type Ethiopian SPA 2020–2021.

Region	Facility	Quality (95%CI)				
		H	HC	HP	C	Subtotal
Afar	34	96.43*	91.43 (81.49,100.00)	58.1 (43.92,72.27)	N/A	77.31 (67.94,86.69)
Amhara	122	92.01 (89.69,94.33)	93.07 (54.38,73.71)	64.05 (43.59,59.54)	N/A	85.42 (81.93,88.91)
Oromia	151	89.84 (87.13,92.54)	90.07 (86.73,93.41)	51.56 (43.59,59.54)	N/A	81.79 (78.37,85.21)
Somali	65	93.45 (87.19,99.71)	87.09 (79.70,94.48)	72.80 (61.88,83.72)	85.71*	82.53 (76.97,88.08)
Benshangule	36	91.43*	96.67 (94.62,98.71)	85.24 (72.33,98.14)	100*	91.27 (85.82,96.72)
SNNPR	211	87.77 (85.62,89.92)	83.10 (78.51,87.68)	51.66 (46.16,57.15)	85.71*	74.61 (71.42,77.81)
Gambela	38	87.14*	89.10 (82.57,95.63)	77.04 (62.92,91.16)	N/A	84.4 (77.82,90.97)
Harari	32	92.86*	93.75*	88.93 (82.11,95.74)	N/A	90.63 (86.17,95.08)
Addis Abeba	43	94.81 (91.00,98.61)	96.26 (91.26,100.00)	N/A	N/A	95.51 (92.52,98.51)
Dire Dawa	42	87.14*	94.76 (90.68,98.85)	70.75 (59.54,81.96)	100*	81.97 (75.30, 88.65)
National level	774	90.33 (89.07,91.59)	89.81 (88,03,91.59)	64.14 (60.78,67.50)	91.43 (79.86,100)	81.98 (80.44 83.51 )

SNNPR: Southern Nations, Nationalities and Peoples; * Small observation (<10); H: Hospitals; HC: Health center; HP: Health post; C: Clinic; N/A: None Available.

**Fig. 2:**
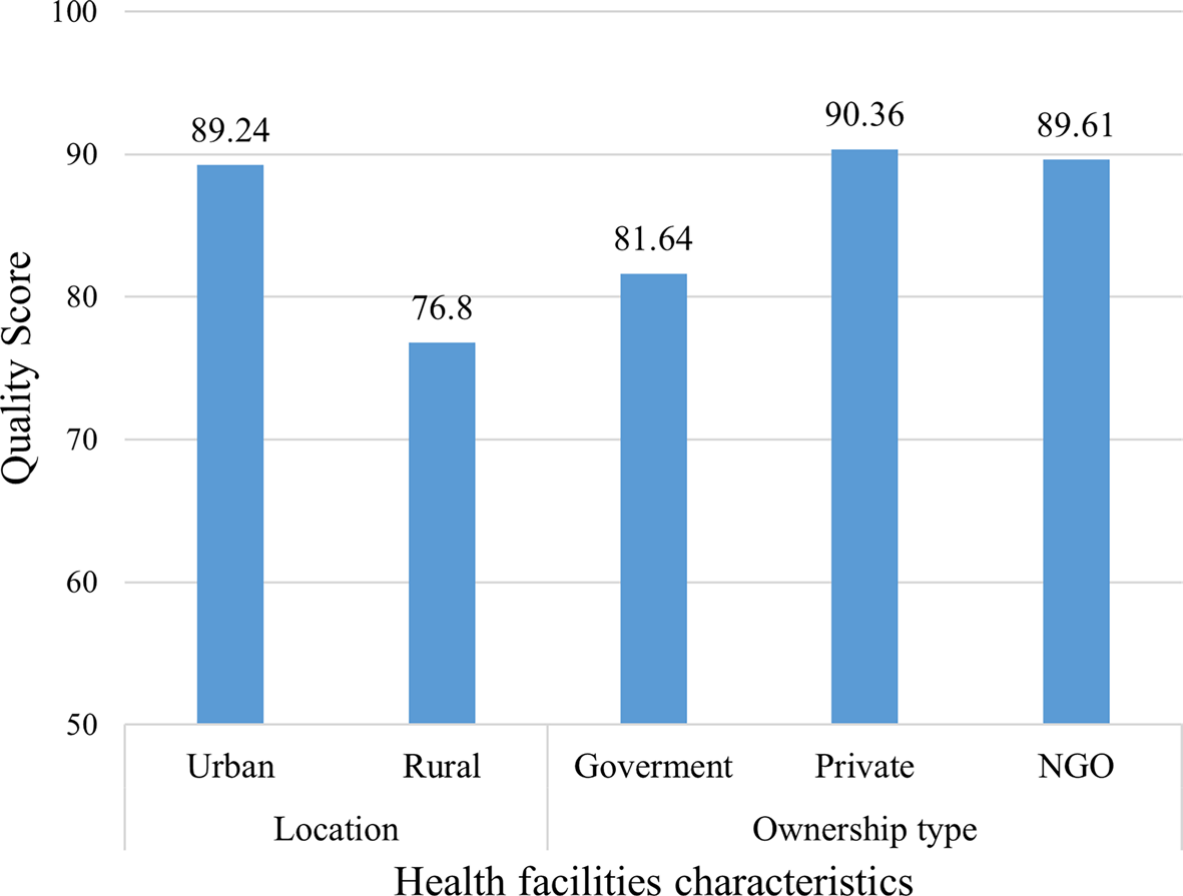
Immunisation service quality by health facility characteristics.

According to areas of health facilities, facilities in urban areas had higher quality (89% (95%CI: 87.65, 90.83)) than the facilities in rural areas (77% (95%CI:74.55,79.06)). Moreover, the quality of immunisation varies with the management authority of Health facilities, private facilities had the highest quality (90% (95% CI: 85.35, 95.36)) and government facilities score the lowest quality (82% (95% CI: 80.06, 83.22)) (Fig. 2).

### Effective coverage

The national effective coverage of immunisation was 34.3% (95% CI: 32.99, 35.50) with a large variation in different regions, ranging from 14.8% (95%CI: 13.03,16.63) in Afar to 83.5% (95%CI: 80.85,86.09) in Addis Ababa. Addis Ababa had the highest effective coverage of immunisation service 83.5% (95%CI: 80.85,86.09) with a 3.9% difference from its crude coverage (87.4% (95%CI: 75.67,99.12)) (Table 5). Sensitivity analyses results were provided in a supplementary file (Supplementary file 1).

**Table 5:** Effective coverage of immunisation service in Ethiopia.

Region	Crude coverage	EC by region	CC-EC (% point)
Afar	19.18 (0.00,41.74)	14.83 (13.03,16.63)	4.35
Amhara	52.43 (45.75,59.12)	44.79 (42.96,46.62)	7.64
Oromia	35.86 (31.17,40.55)	29.33 (28.10,30.56)	6.53
Somali	24.04 (12.47,35.60)	19.84 (18.50,21.17)	4.2
Benshangule	45.66 (9.40,81.92)	41.68 (39.19,44.17)	3.98
SNNPR	29.60 (23.20,35.99)	22.08 (21.14,23.03)	7.52
Gambela	54.36*	45.89 (42.31,49.46)	8.47
Harari	49.92*	45.24 (43.01,47.46)	4.68
Addis Abeba	87.39 (75.67,99.12)	83.47 (80.85,86.09)	3.92
Dire Dawa	54.60*	44.76 (41.17, 48.40)	9.84
National level	39.59 (36.47, 42.70)	34.25 (32.99, 35.50)	5.34

SNNPR: Southern Nations, Nationalities and Peoples; * Small observation (< 10); EC: Effective coverage; CC: Crude coverage.

## Discussion

The effective coverage of child immunisation was 34.3% with a large variation in different regions, ranging from 14.8% in Afar to in Addis Ababa. Addis Ababa had the highest effective coverage of immunisation service 83.5% with a 3.9% difference from its crude coverage (87.4%). This might be due to a geographical variation, distance from the health institution, and a lack of health care infrastructure, particularly in Afar and Somali regions characterized by pastoralist nomadic people. Another cause might be the high population density, particularly in an urban administrative metropolis like Addis Ababa, as well as maternal education, which promotes knowledge about the necessity of immunisation, and educated mothers pick health care facilities that result in better health for their children.

The crude coverage of immunisation services was 39.6%. This showed that despite the efforts to increase the quality of immunisation services, there is a huge gap in both the crude and effective coverage of immunisation services. This finding was lower than a study conducted in Nepal with 90%^34^ crude coverage of all the childhood vaccines, in Mexico with 51% and in Nicaragua with 70%^35^, in India with 76%^36^, in kenya with 66.6%^37^, and in Senegalese with 70.96%^38^. The discrepancy might be due to socio-demographic, economic, accessibility, and availability of health infrastructure and other commodities. The other possible explanation might be due to the educational status of the mother, who has great awareness of the time, the importance, and the side effects of vaccination^39^.

This study also assessed the overall score for the quality of immunisation service (service readiness.) which was 82%. This finding was lower than a study conducted in North Gondar Zone (90.3%)^39^. There was a discrepancy in the overall quality of immunisation service among regions, areas, and types of health facilities, city administrations, and the management authority of Health facilities.

The quality of child immunisation services was often influenced by the availability of key components such as trained staff, medicines, and necessary equipment. This study showed that 43.38% of health facilities had adequate staff and guideline, with significant regional disparities ranging from 36.37% in the Southern Nations, Nationalities, and Peoples’ Region (SNNPR) to 61.63% in Addis Ababa. Additionally, 79.15% of facilities had access to necessary medicines and commodities, with Addis Ababa having the highest rate (92.56%) compared to Afar (67.65%). Moreover, 87.71% of health facilities were adequately equipped, with regional variations from 81.72 to 97.67% in Addis Ababa. These findings aligned with other studies that highlight regional inequalities in healthcare service delivery, particularly in immunization^12,40,41^. This might be due to urban centers like Addis Ababa consistently perform better than rural regions due to resource availability and infrastructure. Studies also showed that resourced health systems ensure the effectiveness of immunisation programs and reducing vaccine-preventable diseases^42^.

The discrepancy among regional level, the quality of immunisation service ranged from 74.6% in SNNPE to 95.5% in Addis Ababa. This might be due to the availability of trained staff in urbanized cities, accessibility, and adequate availability of equipment and commodities. Across areas and types of health facilities, the quality of immunisation services also varied. According to the area of health facilities, facilities in urban areas had higher quality (89.2%) than that of the facilities in rural areas (76.8%). This might be because of easy availability and accessibility of health infrastructures, equipment, and medical supplies and commodities^43,44^.

On the type of health facilities, clinics had the highest quality (91.4%) while health posts had lower quality 64.1%. The possible explanation might be high or medium-level health facilities had updated and well-trained staff, who possess the skill competencies needed to provide child immunisation, while in low-level facilities like health posts service training is not easily available and accessible^45^. Another explanation might be better accessibility and availability of resources, including human power^46^.

### Strength and limitation

This study had many strengths, of this, we used the DHS dataset which had a large sample size. We used the recent EDHS dataset 2019. The study also had limitations, the first limitation is that we used the facility readiness (structural component) to adjust the effective coverage of immunisation, other components were not included. The second limitation was that we used regions to match the dataset, people might get the service from other region facilities and it may cause misclassification. The last limitation was that the Tigray region was not included as the SPA dataset had no record for facilities in Tigray.

## Conclusion

The study revealed that there was low effective coverage of immunisation in Ethiopia. The effective coverage was lower in the Afar and Somali regions. Government facilities and facilities in rural areas had lower-quality immunisation services. Facilities in SNNPE and Afar region had low-quality immunisation. Policymakers and responsible bodies should focus on improving the quality of government facilities and the facilities in rural areas and the SNNPE and Afar region. Additionally, stakeholders could increase the number of trained staff and guidelines across all regions, as well as improve the availability of medicines and commodities, particularly in areas with low supply, such as Afar.

## Electronic supplementary material

Below is the link to the electronic supplementary material.

Supplementary Material 1

Supplementary Material 2

## Data Availability

The dataset is available on the DHS website: https://dhsprogram.com/.

## References

[1] Ebrahim, Y. & Salgedo, W. B. Childhood immunization coverage in Tehulederie district, Northeast of Ethiopia: A community based cross sectional study. *Int. J. Curr. Res.* **7** (9), 20234–20240 (2015).

[2] Al-Gburi, S., Kadhim, H. & Ghazi, H. Association of CD46 cellular receptor gene SNP in measles vaccine response. *Iraqi JMS* **18**(1), 39–46 (2020).

[3] Geweniger, A. & Abbas, K. M. Childhood vaccination coverage and equity impact in Ethiopia by socioeconomic, geographic, maternal, and child characteristics. *Vaccine* **38** (20), 3627–3638 (2020). 32253099 10.1016/j.vaccine.2020.03.040 PMC7171468

[4] General, A. *United Nations Transforming Our World: The 2030 Agenda for Sustainable Development* (Division for Sustainable Development Goals, 2015).

[5] Peck, M. et al. Global routine vaccination coverage, 2018. *Morb. Mortal. Wkly Rep.* **68** (42), 937 (2019). 10.15585/mmwr.mm6842a1 PMC6812836 31647786

[6] Shen, A. K., Fields, R. & McQuestion, M. The future of routine immunization in the developing world: challenges and opportunities. *Global Health: Sci. Pract.* **2** (4), 381–394 (2014). 10.9745/GHSP-D-14-00137 PMC4307855 25611473

[7] Ethiopian Public Health. *Institute (EPHI) and ICF FRR, Maryland: EPHI and ICF*. (Ethiopia Mini Demographic and Health Survey, 2019).

[8] Belachew, E. & Wakgari, D. Factors associated with complete immunization coverage in children aged 12–23 months in Ambo woreda, central Ethiopia. *BMC Public. Health* **12**(566). (2012). 10.1186/1471-2458-12-566 PMC3508824 22839418

[9] Gurmu, E. & Etana, D. Factors influencing children’s full immunization in Ethiopia. *Afr. Popul. Stud.* **30**(2). (2016).

[10] CSA I. *Ethiopia Demographic and Health Survey: Key Indicators Report* (Central Statistical Agency, 2016).

[11] Hussen, A., Bogale, A. L., Ali, J. H. & Haidar, J. Parental satisfaction and barriers affecting immunization services in rural communities: evidence from North Ethiopia. *Sci. J. Public. Health*. **4** (5), 408–414 (2016).

[12] Salah, A. A., Baraki, N., Egata, G. & Godana, W. Evaluation of the quality of expanded program on immunization service delivery in primary health care institutions of Jigjiga zone Somali region, Eastern Ethiopia. *Eur. J. Prev. Med.* **3** (4), 117–123 (2015).

[13] GebreEyesus, F. A., Assimamaw, N. T., GebereEgziabher, N. T. & Shiferaw, B. Z. Maternal satisfaction towards childhood immunization service and its associated factors in Wadla district, North Wollo, Ethiopia, 2019. *Int. J. Pediatr.* **2020**, 1–13 (2020).

[14] Tanahashi, T. Health service coverage and its evaluation. *Bull. World Health Organ.* **56** (2), 295 (1978). 96953 PMC2395571

[15] Penchansky, R. & Thomas, J. W. The concept of access: definition and relationship to consumer satisfaction. Med. Care. :127 – 40. (1981). 10.1097/00005650-198102000-00001 7206846

[16] Adokiya, M. N., Baguune, B. & Ndago, J. A. Evaluation of immunization coverage and its associated factors among children 12–23 months of age in Techiman municipality, Ghana, 2016. *Archives Public. Health*. **75**, 1–10 (2017). 10.1186/s13690-017-0196-6 PMC5483840 28652913

[17] Animaw, W., Taye, W., Merdekios, B., Tilahun, M. & Ayele, G. Expanded program of immunization coverage and associated factors among children age 12–23 months in Arba minch town and Zuria district, Southern Ethiopia, 2013. *BMC Public. Health*. **14** (1), 1–10 (2014). 24884641 10.1186/1471-2458-14-464 PMC4032449

[18] Gualu, T. & Dilie, A. Vaccination coverage and associated factors among children aged 12–23 months in debre markos town, Amhara regional state, Ethiopia. *Adv. Public Health* **2017** (2017).

[19] Timane, A. J., Oche, O. M., Umar, K. A., Constance, S. E. & Raji, I. A. Clients’ satisfaction with maternal and child health services in primary health care centers in Sokoto Metropolis, Nigeria. *Edorium J. Maternal Child. Health*. **2**, 9–18 (2017).

[20] Tesema, G. A., Tessema, Z. T., Tamirat, K. S. & Teshale, A. B. Complete basic childhood vaccination and associated factors among children aged 12–23 months in East Africa: a multilevel analysis of recent demographic and health surveys. *BMC Public. Health*. **20** (1), 1–14 (2020). 33256701 10.1186/s12889-020-09965-y PMC7708214

[21] Dametie, D. *Quality of Expanded Program of Immunization and Associated Factors with Client Satisfaction in Shashemene Woreda, Oromia Region* (Jimma University, 2017).

[22] Tesfaye, E., Debie, A., Sisay, F. & Tafere, T. Z. Maternal satisfaction on quality of childhood vaccination services and its associated factors at public health centers in addis Ababa, Ethiopia. *BMC Health Serv. Res.* **23** (1), 1315 (2023). 38031017 10.1186/s12913-023-10174-7 PMC10685558

[23] Amare, G. et al. Vaccine safety practices and its implementation barriers in Northwest Ethiopia: A qualitative study. *Ethiop. J. Health Dev.* **35**(3). (2021).

[24] Haile, T. G., Asefa, A., Mirkuzie, A. & Benova, L. (eds) Effective coverage of curative child health services in Ethiopia? Tracking progress towards universal health coverage. In *33rd EPHA Annual Conference* (2022).

[25] Marsh, A. D. et al. Effective coverage measurement in maternal, newborn, child, and adolescent health and nutrition: progress, future prospects, and implications for quality health systems. *Lancet Global Health*. **8** (5), e730–e6 (2020). 32353320 10.1016/S2214-109X(20)30104-2 PMC7196884

[26] Organization, W. H. *Primary Health Care Systems (Primasys) Case Study from Ethiopia: Abridged Version*. (World Health Organization, 2017).

[27] Croft, T. N. et al. *Guide to DHS Statistics*.Vol. 645 (ICF, 2018).

[28] Donabedian, A. The quality of care: how can it be assessed? *Jama* **260** (12), 1743–1748 (1988). 3045356 10.1001/jama.260.12.1743

[29] Wang, W., Mallick, L., Allen, C. & Pullum, T. Effective coverage of facility delivery in Bangladesh, Haiti, Malawi, Nepal, Senegal, and Tanzania. *PloS One*. **14** (6), e0217853 (2019). 31185020 10.1371/journal.pone.0217853 PMC6559642

[30] EPHI W. *Ethiopia: Services Availability and Readiness Assessment Summary Report Ethiopia Service Availability and Readiness Assessment 2016: Summary Report* (International institute for Primary Health Care Ethiopia, 2016).

[31] Tegegne, T. K., Chojenta, C., Getachew, T., Smith, R. & Loxton, D. Service environment link and false discovery rate correction: methodological considerations in population and health facility surveys. *PLOS ONE*. **14** (7), e0219860 (2019). 31318939 10.1371/journal.pone.0219860 PMC6638937

[32] Melaku Birhanu, A. et al. Low effective coverage of HIV testing and counselling services during antenatal care in Ethiopia: evidence from the demographic and health survey and service provision assessment. *BMJ Public. Health*. **2** (2), e001158 (2024). 40018594 10.1136/bmjph-2024-001158 PMC11816946

[33] Shengelia, B., Tandon, A., Adams, O. B. & Murray, C. J. Access, utilization, quality, and effective coverage: an integrated conceptual framework and measurement strategy. *Soc. Sci. Med.* **61** (1), 97–109 (2005). 15847965 10.1016/j.socscimed.2004.11.055

[34] Rauniyar, S. K., Iwaki, Y., Yoneoka, D., Hashizume, M. & Nomura, S. Age-appropriate vaccination coverage and its determinants in children aged 12–36 months in Nepal: a National and subnational assessment. *BMC Public. Health*. **21**, 1–12 (2021). 34758802 10.1186/s12889-021-11841-2 PMC8582094

[35] Colson, K. E. et al. Comparative estimates of crude and effective coverage of measles immunization in low-resource settings: findings from Salud Mesoamérica 2015. *PloS One*. **10** (7), e0130697 (2015). 26136239 10.1371/journal.pone.0130697 PMC4489764

[36] Summan, A., Nandi, A., Schueller, E. & Laxminarayan, R. Public health facility quality and child immunization outcomes in rural India: A decomposition analysis. *Vaccine* **40** (16), 2388–2398 (2022). 35305825 10.1016/j.vaccine.2022.03.017 PMC8996686

[37] Mutua, M. K. et al. Fully immunized child: coverage, timing and sequencing of routine immunization in an urban poor settlement in Nairobi, Kenya. *Trop. Med. Health*. **44**, 1–12 (2016). 27433132 10.1186/s41182-016-0013-x PMC4940963

[38] Sarker, A. R., Akram, R., Ali, N., Chowdhury, Z. I. & Sultana, M. Coverage and determinants of full immunization: vaccination coverage among Senegalese children. *Medicina* **55** (8), 480 (2019). 31416213 10.3390/medicina55080480 PMC6723170

[39] Delele, T. G., Biks, G. A., Abebe, S. M. & Kebede, Z. T. Essential newborn care service readiness and barriers in Northwest Ethiopia: A descriptive survey and qualitative study. *J. Multidiscip. Healthc.* 713–725 (2021). 10.2147/JMDH.S300362 PMC8001582 33790570

[40] Geremew, T. T., Gezie, L. D. & Abejie, A. N. Geographical variation and associated factors of childhood measles vaccination in Ethiopia: a Spatial and multilevel analysis. *BMC Public. Health*. **19**, 1–15 (2019). 31470822 10.1186/s12889-019-7529-z PMC6716824

[41] Awol, M., Alemu, Z. A., Moges, N. A. & Jemal, K. Geographical variations and associated factors of defaulting from immunization among children aged 12 to 23 months in Ethiopia: using Spatial and multilevel analysis of 2016 Ethiopian demographic and health survey. *Environ. Health Prev. Med.* **26** (1), 65 (2021). 34118886 10.1186/s12199-021-00984-8 PMC8199811

[42] Hayir, T. M. M., Magan, M. A., Mohamed, L. M., Mohamud, M. A. & Muse, A. A. Barriers for full immunization coverage among under 5 years children in Mogadishu. *Somalia J. Family Med. Prim. Care*. **9** (6), 2664–2669 (2020). 32984104 10.4103/jfmpc.jfmpc_119_20 PMC7491846

[43] Weeks, W. B. et al. Rural-urban disparities in health outcomes, clinical care, health behaviors, and social determinants of health and an action-oriented, dynamic tool for visualizing them. *PLOS Global Public. Health*. **3** (10), e0002420 (2023). 37788228 10.1371/journal.pgph.0002420 PMC10547156

[44] Asresie, M. B. & Arora, A. Urban-rural disparities and change in postnatal care use from 2016 to 2019 in Ethiopia: multivariate decomposition analysis. *PLOS ONE*. **19** (9), e0299704 (2024). 39226258 10.1371/journal.pone.0299704 PMC11371223

[45] Manyazewal, T. et al. Improving immunization capacity in Ethiopia through continuous quality improvement interventions: a prospective quasi-experimental study. *Infect. Dis. Poverty*. **07** (06), 35–48 (2018). 10.1186/s40249-018-0502-8 PMC6267782 30497515

[46] Tilahun, B. et al. What we know and don’t know about the immunization program of Ethiopia: a scoping review of the literature. *BMC Public. Health*. **20** (1), 1365 (2020). 32894099 10.1186/s12889-020-09304-1 PMC7487697

