# Supplementary material for: Effective coverage of child immunisation service in Ethiopia
Source: Sci Rep. 2025 May 29;15:18938. doi: 10.1038/s41598-025-02885-0 (PMC12122664; doi:10.1038/s41598-025-02885-0)
Supplement: Supplementary file 1 — Supplementary Material 1 [file 41598_2025_2885_MOESM1_ESM.docx]

Table S1 1: Indicators of child immunization service readiness (SARA 2016)

| **Staff and guideline** | |
| --- | --- |
| 1 | Received any training on child vaccination |
| 2 | Availability of national guidelines for child vaccinations |
| **Medicine and commodities** | |
| 1 | DPT+hepb+Hib (pentavalent) |
| 2 | Oral polio vaccine |
| 3 | Measles vaccine and diluent |
| 4 | BCG vaccine and diluent |
| 5 | Pneumococcal conjugate vaccine |
| **Equipment** | |
| 1 | Auto-disable syringes with needles/single-use standard disposable s |
| 2 | Register or tally sheets |
| 3 | Vaccine storage in service area: refrigerator |
| 4 | Temperature of vaccine storage in service area |
| 5 | Blank immune (child) cards or health passport |
| 6 | Ice packs for vaccine carriers in service area |
| 7 | Sharps box |
