# Supplementary material for: Effective coverage of child immunisation service in Ethiopia
Source: Sci Rep. 2025 May 29;15:18938. doi: 10.1038/s41598-025-02885-0 (PMC12122664; doi:10.1038/s41598-025-02885-0)
Supplement: Supplementary file 2 — Supplementary Material 2 [file 41598_2025_2885_MOESM2_ESM.docx]

**Sensitivity analysis**

The sensitivity analysis reveals that excluding specific regions has a significant effect on the quality and effective coverage results. When the Southern Nations, Nationalities, and Peoples' Region (SNNP) is excluded, the crude coverage increases to 42.24% (95% CI: 38.71, 45.77), quality improves to 84.74% (95% CI: 83.05–86.42), and effective coverage rises to 38.81% (95% CI: 37.28, 40.33). Excluding Oromia results in a crude coverage of 42.36% (95% CI: 38.20–46.52), quality at 82.02% (95% CI: 80.30, 83.74), and effective coverage of 35.44% (95% CI: 33.92–36.96) (Table S2).

Table S2: Sensitivity analysis of effective coverage of child immunization by region

| Region | Crude coverage (95%CI) | Quality (95%CI) | Effective coverage (95%CI) |
| --- | --- | --- | --- |
| All regions | 39.59 (36.47, 42.70) | 81.98 (80.44, 83.51) | 34.25 (32.99, 35.50) |
| Region (- SNNP) | 42.24 (38.71, 45.77) | 84.74 (83.05, 86.42) | 38.81 (37.28, 40.33) |
| Region (-Oromia) | 42.36 (38.20, 46.52) | 82.02 (80.30, 83.74) | 35.44 (33.92, 36.96) |
| Region (-Amhara) | 35.77 (32.29, 39.25) | 81.33 (79.63, 83.03) | 32.27 (30.87, 33.68) |
